# The Management after Operation, for Lacerations of Perineum Extending through the Sphincter Ani Muscle

**Published:** 1885-04

**Authors:** George H. Noble

**Affiliations:** Atlanta, Ga.


### THE MANAGEMENT AFTER OPERATION, FOR LACERATIONS OF PERINEUM EXTENDING THROUGH THE SPHINCTER ANI MUSCLE.

BY GEORGE H. NOBLE, M. D., ATLANTA, GA.

The want of better success in this operation is generally due either to a failure in the approximation of the ends of the sphincter, or to a proper after-treatment. The first being well set forth in the more recent works on gynecology, I shall speak of the latter only.

My object is to give a mode of management satisfactorily employed by myself in our Infirmary, which I trust will add to the success in the hands of others.

The usual preparatory treatment, consisting of free daily evacuation of the bowels for some seven or ten days prior to the closure of the rent, and feeding the patient upon soups, etc., for the same period, is essential to success.

After the operation I introduce a catheter and effect the escape of gaseous collections as often as the slightest sense of distention is felt in the rectum, which may not be more than once in two days, but daily, if necessary.

The finger should be placed upon the verge of the anus and pressed in the direction of the cocyx, that the instrument may not come in contact with the wire suture.

On the fourth or fifth day after operation I introduce in like manner one of my rectal irrigation and enemata tubes, through which is injected a quantity of warm water strongly impregnated with inspissated ox gall; but as soon as any distention is felt on the part of the patient, or as soon as the bowel is inclined to contract, it is allowed to return through the tube and empty into a vessel on the floor. This may be repeated a number of times without removal of the instrument, until the bowel is thoroughly emptied of all fœcal matter.

The use of the catheter should be continued, and on the eighth or tenth day the above procedure is again resorted to, when (eighth day) the sutures are removed in part or entire, as may be desired.

The twelfth or fourteenth day is as early as any solid food should be allowed, and that of the most digestible character.

At this juncture a dose of castor oil is required in sufficient quantity to cause free liquid evacuations from the bowels.

From this time on daily stools, soft in consistency, should be encouraged, and the patient permitted to leave the bed.

In addition to the above, a solution of one of the bromides (preferably ammonium), with a small quantity of morphia sulphate, should be used to secure rest and to control the bowels.

The tube and its employment in these cases are original with me, and it was the advantages derived therefrom that prompted this little article.

By it we do away with the unpleasantness of the bed pan and avoid the danger of tearing open the wound, also of the repeated introductions of the syringe nozzle, which otherwise becomes necessary. It relieves all tension, and the action of the sphincter muscle in the removal of the fœcal matter, while the use of the catheter explains itself.

